# Programmed death-ligand 1 overexpression is a prognostic marker for aggressive papillary thyroid cancer and its variants

**DOI:** 10.18632/oncotarget.8698

**Published:** 2016-04-12

**Authors:** Subrata Chowdhury, Joe Veyhl, Fatima Jessa, Olena Polyakova, Ahmed Alenzi, Christina MacMillan, Ranju Ralhan, Paul G. Walfish

**Affiliations:** ^1^ Alex and Simona Shnaider Research Laboratory in Molecular Oncology, Mount Sinai Hospital, Toronto, Ontario, Canada; ^2^ Joseph and Mildred Sonshine Family Centre for Head and Neck Diseases, Department of Otolaryngology-Head and Neck Surgery Program, Mount Sinai Hospital, Toronto, Ontario, Canada; ^3^ Department of Pathology and Laboratory Medicine, Mount Sinai Hospital, Toronto, Ontario, Canada; ^4^ Laboratory Medicine and Pathobiology, University of Toronto, Toronto, Ontario, Canada; ^5^ Department of Otolaryngology-Head and Neck Surgery, Mount Sinai Hospital, Toronto, Ontario, Canada; ^6^ Department of Otolaryngology-Head and Neck Surgery, University of Toronto, Toronto, Ontario, Canada; ^7^ Department of Medicine, Endocrine Division, Mount Sinai Hospital and University of Toronto Medical School, Toronto, Ontario, Canada

**Keywords:** thyroid cancer, benign nodule, programmed death-ligand 1, protein biomarkers, subcellular localization

## Abstract

Programmed death-ligand 1(PD-L1) expression on tumor cells is emerging as a potential predictive biomarker in anti-PD-L1 directed cancer immunotherapy. We analyzed PD-L1 expression in papillary thyroid carcinoma (PTC) and its variants and determined its prognostic potential to predict clinical outcome in these patients. This study was conducted at an academic oncology hospital which is a prime referral centre for thyroid diseases. Immunohistochemical subcellular localization (IHC) analyses of PD-L1 protein was retrospectively performed on 251 archived formalin fixed and paraffin embedded (FFPE) surgical tissues (66 benign thyroid nodules and 185 PTCs) using a rabbit monoclonal anti-PD-L1 antibody (E1L3N, Cell Signaling Technology) and detected using VECTASTAIN rapid protocol with diaminobenzidine (DAB) as the chromogen. The clinical-pathological factors and disease outcome over 190 months were assessed; immunohistochemical subcellular localization of PD-L1 was correlated with disease free survival (DFS) using Kaplan Meier survival and Cox multivariate regression analysis. Increased PD-L1 immunostaining was predominantly localized in cytoplasm and occasionally in plasma membrane of tumor cells. Among all combined stages of PTC, patients with increased PD-L1 membrane or cytoplasmic positivity had significantly shorter median DFS (36 months and 49 months respectively) as compared to those with PD-L1 negative tumors (DFS, both 186 months with *p* < 0.001 and *p* < 0.01 respectively). Comparison of PD-L1^+^ and PD-L1^−^ patients with matched staging showed increased cytoplasmic positivity in all four stages of PTC that correlated with a greater risk of recurrence and a poor prognosis, but increased membrane positivity significantly correlated with a greater risk of metastasis or death only in Stage IV patients. In conclusion, PD-L1 positive expression in PTC correlates with a greater risk of recurrence and shortened disease free survival supporting its potential application as a prognostic marker for PTC.

## INTRODUCTION

Thyroid carcinoma (TC) is the most common malignant endocrine tumor with more than 500,000 diagnosed cases in the United States and one of the most rapidly increasing malignancies in both men and women [1]. The estimated new cases and deaths in 2015 from TC are 62,450 (3.8% of all the cancer cases; 15,220 men and 47,230 women) and 1,950 (0.3% of all cases; 870 men and 1080 women) respectively [2]. Most of the thyroid cancers (well differentiated papillary and follicular TCs) have excellent prognosis if detected early and treated appropriately. The overall five-year survival rate of patients with thyroid cancer is about 97.9% [2]. However, while anaplastic TC accounts for only 2% of all the cases, 50% of the deaths are from this malignancy annually and the remainder due to aggressive variants of metastatic papillary and follicular TCs which have higher risk of recurrence, shortened disease free survival and death [3, 4]. The localized differentiated thyroid cancer (DTC) is managed by surgery and radioiodine therapy; about 10% of these patients develop progressive invasive primary disease and 5% have distant metastases. About 20–30% of DTC patients have recurrence, often in locoregional lymph nodes, which may require aggressive surgical neck dissection and radiotherapy [5]. The early stratification of patients with poor prognosis would enable oncologists to choose an appropriate treatment strategy thereby improving survival and quality of life. Biomarker(s) that serve as a tool for timely detection and intervention could direct effective adjuvant treatment to those patients who require it and spare patients with non-aggressive PTCs from unnecessary follow up excess investigational procedures and the concern about possible potential surgery and radiation therapy in the future. The Cancer Genome Atlas (TCGA) project has yielded new biological insights in different cancer types including TC through generating genomic, transcriptomic, epigenomic and proteomic data. However, the potential clinical utility of these data in aggregate remains largely unknown [6]. The lack of universally accepted protein biomarkers to define the aggressive PTC poses a major challenge in predicting disease prognosis and management.

Understanding the importance of immune checkpoints in blocking tumor recognition has led to development of novel immunotherapies targeting the cytotoxic T-lymphocyte-associated protein (CTLA-4, CD 152), the programmed cell death protein-1 (PD-1, CD279) and its ligand programmed death ligand 1 (PD-L1, CD274) [7]. PD-L1 is expressed by B and T cells, monocytes, macrophages and dendritic cells (APCs) [8] and play important role in regulating immune responses [9–12]. Moreover, over-expression of PD-L1 in a variety of tumors and *in-vitro* experimental models indicate compromise of immune surveillance mechanism for cancer cells in the tumor microenvironment, by interaction with PD-1 [13]. PD-L1 containing tumor cells can induce T cell apoptosis, IL-10 production and can protect tumor cells from lysis by cytotoxic T lymphocytes (CTLs) [14].

PD-L1 overexpression has been reported in various human cancers, including head and neck, breast, ovarian, renal, pancreas, esophageal, non-small cell lung cancer (NSCLC), melanoma and glioblastoma [15–17] and linked to poor prognosis and increased resistance to anticancer therapies [18]. Recent clinical trials directed against critical immune checkpoint molecules have shown promising antitumor activity in several malignancies [13]. The importance of PD-1/PD-L1 stems from studies showing a restoration of host immunity against tumors and favorable clinical responses. Anti-PD-1 therapy has generated potential clinical benefits by inducing regression of aggressive tumors and improving patient survival in melanoma, bladder, lung and kidney cancers [19]. The search for a predictive biomarker to identify patients who are likely to respond to anti- PD-1/PD-L1 immunotherapy poses an important clinical challenge in view of the observed autoimmune side effects from agents targeting this axis [20].

However, different factors can contribute in determining the response to PD-L1 targeted therapy, including the presence of a tumor-specific T cell response and other immunogenetic and environmental factors [21]. Immunotherapies against immune checkpoints that inhibit T cell activation (CTLA-4 and PD-1/PD-L1 axis) are emerging as promising treatments for several metastatic malignancies. However, the precise adverse effects of these therapies on thyroid gland function and thyroid cancer have not been well described. Our current study was designed to investigate the alterations in expression and sub-compartmental localization of PD-L1 in different stages of PTC compared to benign nodular goiter to determine its potential as a prognostic marker for this malignancy.

## RESULTS

Among the patients, 80% were females with the median age of diagnosis in patients with benign thyroid nodules being 47 years (range 17–80 years); while the median age of PTC patients was 45 years (18–85 years) (Table 1). Of the 185 PTC cases, 124 (67%) showed multifocality, 95 (51%) tumors were classified as encapsulated and 88 (45%) tumors had microcarcinoma (Table 2). As per the AJCC classification PTC cases were classified as stage I (63 cases, 34%), II (48 cases, 26%), III (30 cases, 16%) and IV (44 cases, 22%) (Table 2).

**Table 1:** Clinical and pathological parameters of patients in the test and validation sets

Clinicopathological parameters	Total cases (*n* = 251)
**Papillary Thyroid Cancer (PTC)**	**185**
Age	
Range 18–85 years; Median = 45 years	
Gender	
Male	39
Female	146
**Tumor Types**	
Anaplastic TC	18
Insular PTC	9
Poorly Differentiated PTC	2
Classical Variant PTC	90
Follicular Variant PTC	45
Tall Cell Variant PTC	21
**Benign**	**66**
Age	
Range 17–80 years; Median = 47 y	
Gender	
Male	13
Female	53
**Histological Diagnosis**	
Multiple Goiter	21
Hyperplastic Nodules	17
Thyroid Cyst	05
Colloid Nodule	05
Dominant Nodule	04
Graves' Disease	04
*Lymphocytic Thyroiditis*	*05*
*Hashimoto's Thyroiditis*	*05*

**Table 2:** Correlation of PD-L1 expression with clinico-pathological parameters of thyroid carcinoma patients

Clinico-pathologic parameters	Total (*N* = 251)	Cytoplasm positive *n* (%)	Cytoplasm *p* value	Membrane positive *n* (%)	Membrane *p* value
Papillary Thyroid Carcinoma (PTC)	185	123 (66.5%)		74 (40.0%)	
Aggressive PTC	74	71 (95.9%)	0.01^a^	53 (71.6%)	0.001^b^
Non-Aggressive PTC	111	53 (47.7%)		21 (18.9%)	
**Stage**					
I	63	15 (23.8%)		6 (9.5%)	
II	48	37 (77.1%)		15 (31.30%)	
III	30	29 (96.7%)		22 (73.3%)	
IV	44	42 (95.5%)		31 (70.5%)	
**Multifocal**					
No	61	46 (75.4%)	0.35	30 (49.2%)	0.49
Yes	124	79 (63.7%)		44 (35.5%)	
**Microcarcinoma**					
No	95	67 (70.5%)	0.24	27 (28.4%)	0.32
Yes	90	56 (62.2%)		47 (52.2%)	
**Encapsulated**					
No	88	75 (85.2%)	0.13	31 (35.2%)	0.21
Yes	97	48 (49.5%)		43 (44.3%)	
**Perineural Invasion**					
No	181	120 (66.3%)	0.62	71 (39.2%)	0.68
Yes	4	4 (100%)		3 (75%)	
**Benign Nodules**	66				
Lymphocytic Thyroiditis	5	3 (60%)		2 (40%)	
Hashimoto's Thyroiditis	5	4 (80%)		1 (20%)	
Other Benign Nodules	56	5 (17%)		3 (10%)	

a, b *p* value Aggressive vs Non-Aggressive PTC.

### Immunohistochemical analysis of PD-L1 expression in thyroid tissues

Immunohistochemical analysis of PD-L1 was carried out to determine differences in its subcellular localization and expression in benign thyroid tissues and different subtypes of PTC. The positive vs negative cut offs for PD-L1 expression were computed from Receiver operating curves (ROC). The cytoplasmic staining cut off had a sensitivity and specificity of 91% and 85% respectively [with Positive predictive value (PPV) = 92% and Negative predictive value (NPV) = 82%]. For the membrane staining cut off these respective values for sensitivity and specificity were 90% and 85% [with PPV = 88% and NPV = 87%]. From these analyses, the cut-off for PD-L1 increased immunopositivity in the cytoplasm was ≥ 4.5; for the plasma membrane ≥ 2.1 and for combined cytoplasm and membrane immunopositivity the cut-off was ≥ 5.25.

**Figure 1:**
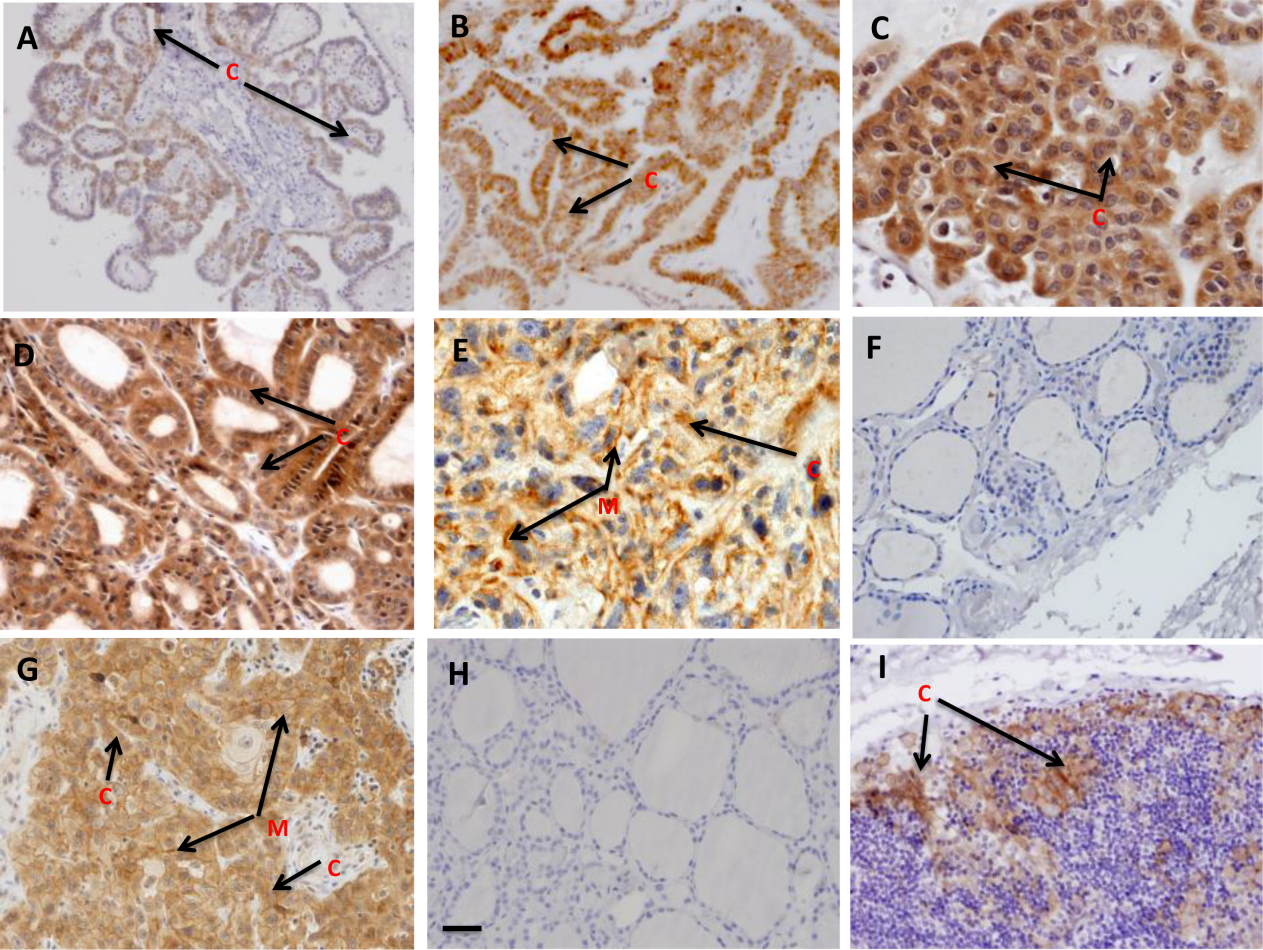
Immunohistochemical analysis of PD-L1 in thyroid tissues: Paraffin embedded sections of different stages of PTC, benign thyroid nodules and concurrent thyroiditis were stained using anti-PD-L1 monoclonal antibody as described in the method sections The representative photomicrographs show immunostaining of PD-L1 in thyroid carcinomas. Faint cytoplasmic staining was observed in early stage (Stage I) of thyroid carcinoma (**A**); Mild cytoplasmic staining was observed in Stage II (**B**); moderate to very strong cytoplasmic staining and mild to moderate membranous staining of PD-L1 were visible in advanced stages (Stage III, IV) of thyroid carcinomas (**C**), (**D**), (**E**). Benign tissues section showing no detectable PD-L1 staining (**F**); Human OSCC tissue as positive control (**G**); Thyroid tissue incubated with isotype specific IgG showing no detectable immunostaining for PD-L1 as Negative control (**H**). Lymphocytic Thyroiditis tissue section showing increased PD-L1 staining mostly cytoplasmic (arrow indicated as C) and occasionally in membrane (M) and presence of increased lymphocytes infiltration (**I**). Original magnification (A–F) and (H): ×200; (G, I): ×400.

Among 185 PTC tissues analyzed, 123 tissues (66.5%) showed cytoplasmic PD-L1 expression, whereas 74 tissues (40.0%) showed membrane localization (Table 2). Among the 74 aggressive PTCs, 71 cases (95.9%) showed cytoplasmic and 53 cases (71.6%) membrane localization, whereas the sub-cellular distribution in the non-aggressive PTCs was 53 (47.7%) and 21 (18.9%) respectively (Table 2). Representative tissue sections showing the sub-cellular localization pattern of PD-L1 protein in benign thyroid nodules and different stages of PTC are shown in Figure 1. Stage I PTCs showed low level of cytoplasmic staining (Figure 1A), while increased predominantly cytoplasmic and low membrane expression was observed in Stage II PTCs (Figure 1B). Differences in patterns of staining were observed in different stages of PTC- Stage III and Stage IV PTC cases showed markedly increased PD-L1 expression (Figure 1C, 1D and 1E). The tall-cell variant PTCs showed strong cytoplasmic and membranous staining of PD-L1. No detectable PD-L1 staining was observed in the benign nodules (Figure 1F).

Among the PTCs, no correlation was observed between PD-L1 expression and multifocality, capsule extension, microcarcinoma and perineural invasion. Importantly, we observed increased PD-L1 expression in benign chronic lymphocytic (Figure 1I) and Hashimoto's thyroiditis. Further, a retrospective analysis of clinical outcome of PTC patients revealed 72 cases (38.9%) cases had recurrence; higher stages patients showed increased recurrence- [Stage I: 6 (3.2%); Stage II: 22 (11.9%); Stage III: 19 (10.7%) and Stage IV: 25 (13.5%)]. Among the stages I, II, III and IV recurrence cases, positive cytoplasmic PD-L1 staining were 8, 24, 21 and 25 respectively whereas the membranous positive PD-L1 staining were 2, 3, 14 and 23 respectively.

**Figure 2:**
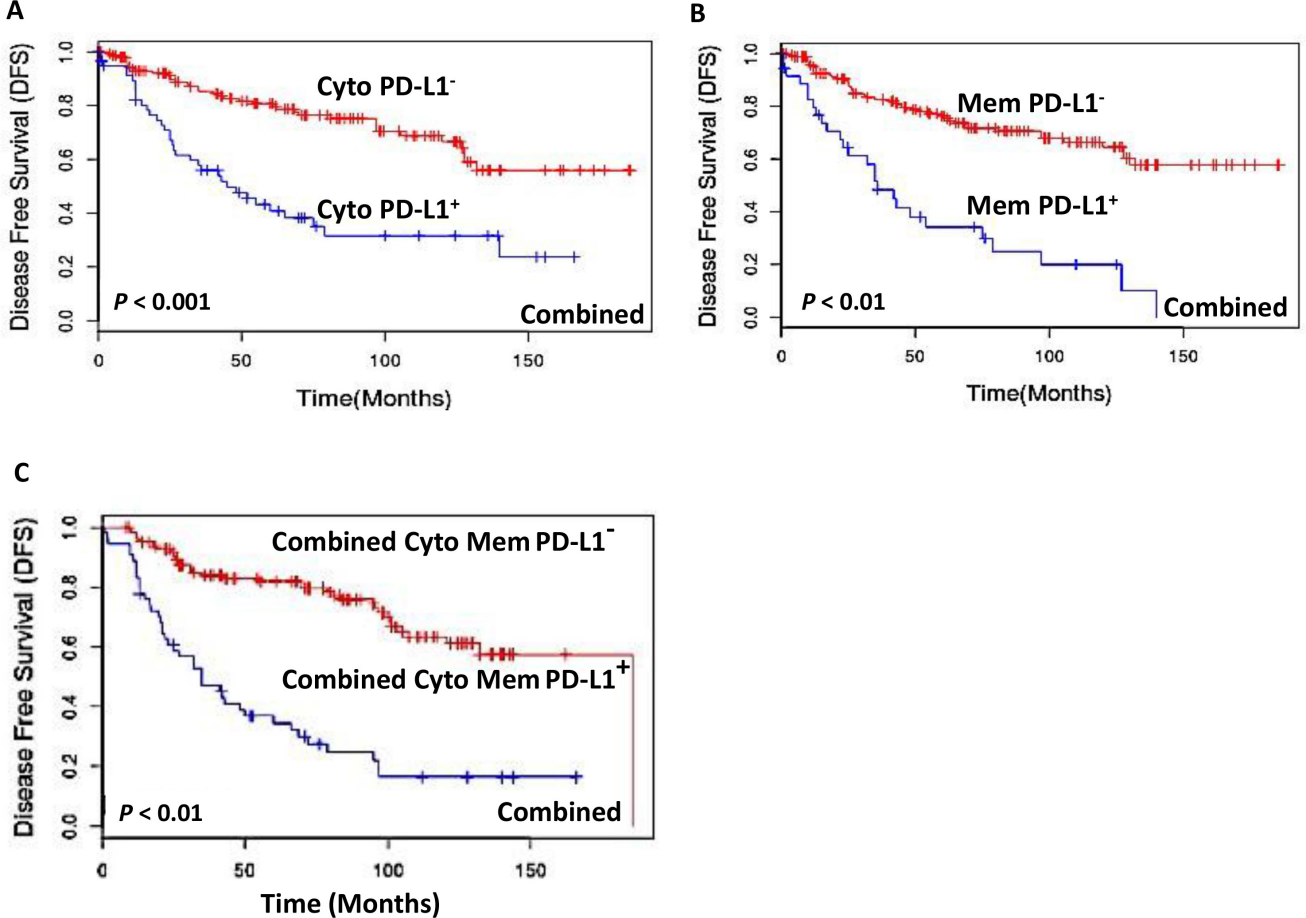
Kaplan-Meier estimation of disease-free survival in PTC patients Disease free survival curves showing PD-L1 expression among all the combined stages of PTCs in: (**A**) cytoplasm [PD-L1 positive median DFS = 49 months (blue lines) and PD-L1 negative DFS = 186 months (red lines); *p* < 0.001]; (**B**) plasma membrane [PD-L1 membrane positive tumors median disease free survival (DFS) = 36 months (blue lines) and PD-L1 negative DFS = 186 months (red lines); *p* < 0.01]; (**C**) Combined cytoplasmic and membrane PD-L1 positive patients [DFS = 42 months (blue lines) and PD-L1 negative [DFS = 186 months (red lines); *p* < 0.01].

Cox-regression (multivariate) analysis was performed to determine the prognostic potential of PD-L1 expression in comparison to clinical and pathological parameters that were independently significant in Kaplan-Meier analysis. The parameters tested were PD-L1 staining in membrane, cytoplasm and combined membrane and cytoplasm staining in patients with different stages of PTC. Disease free survival (DFS) was calculated through Kaplan-Meier analysis for each of the stages. DFS is defined as time period between completion of primary treatment and detection of residual disease or recurrence of the disease or death. In this cohort during the follow up no death was reported in patients with stage I and II. While in stage III and IV, the reported death incidences were 2 and 5 respectively. Kaplan Meier analysis showed that among all the combined stages of PTCs, patients with PD-L1 cytoplasmic positive tumor cells showed significantly reduced median DFS (49 months) as compared to those with PD-L1 negative tumors (median DFS = 186 months; *p* < 0.001) (Figure 2A). PD-L1 membrane positivity was also associated with reduced median DFS of 36 months compared to DFS of 186 months for PD-L1 negative tumors (*p* < 0.01) (Figure 2B). Further, combined PD-L1 cytoplasm and membrane positivity was also associated with reduced median DFS of 42 months as compared to those with PD-L1 negative tumors (DFS = 186 months; *p* < 0.01) (Figure 2C).

**Figure 3:**
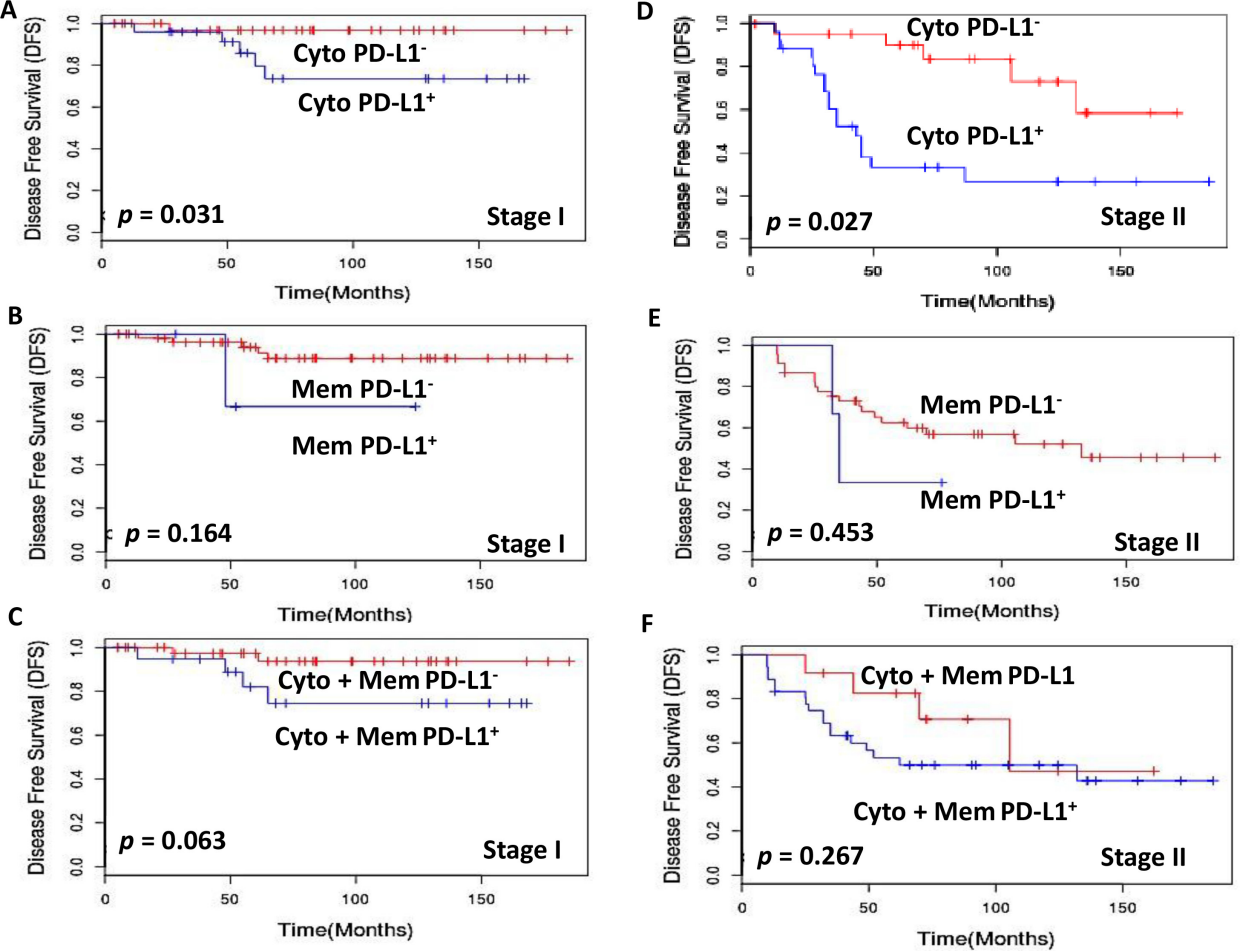
Kaplan-Meier estimation of disease-free survival in stage I and II PTC patients Disease free survival curves showing PD-L1 expression in (**A**) Stage I cytoplasm [PD-L1 positive median disease free survival (DFS) = 166 months (blue lines) and PD-L1 negative DFS = 185 months (red lines); *p* = 0.031]. (**B**) Stage I membrane [PD-L1 positive median disease free survival (DFS) = 124 months (blue lines) and PD-L1 negative DFS = 185 months (red lines); *p* = 0.164]. (**C**) Stage I combined cytoplasm and membrane [PD-L1 positive median disease free survival (DFS) = 168 months (blue lines) and PD-L1 negative DFS = 185 months (red lines); *p* = 0.063]. (**D**) Stage II cytoplasm [PD-L1 positive median disease free survival (DFS) = 62 months (blue lines) and PD-L1 negative DFS = 132 months (red lines); *p* = 0.027]. (**E**) Stage II membrane [PD-L1 positive median disease free survival (DFS) = 62 months (blue lines) and PD-L1 negative DFS = 132 months (red lines); *p* = 0.453]. (**F**) Disease free survival curves showing PD-L1 expression in Stage II combined cytoplasm and membrane [PD-L1 positive median disease free survival (DFS) = 62 months (blue lines) and PD-L1 negative DFS = 105 months (red lines); *p* = 0.267].

**Figure 4:**
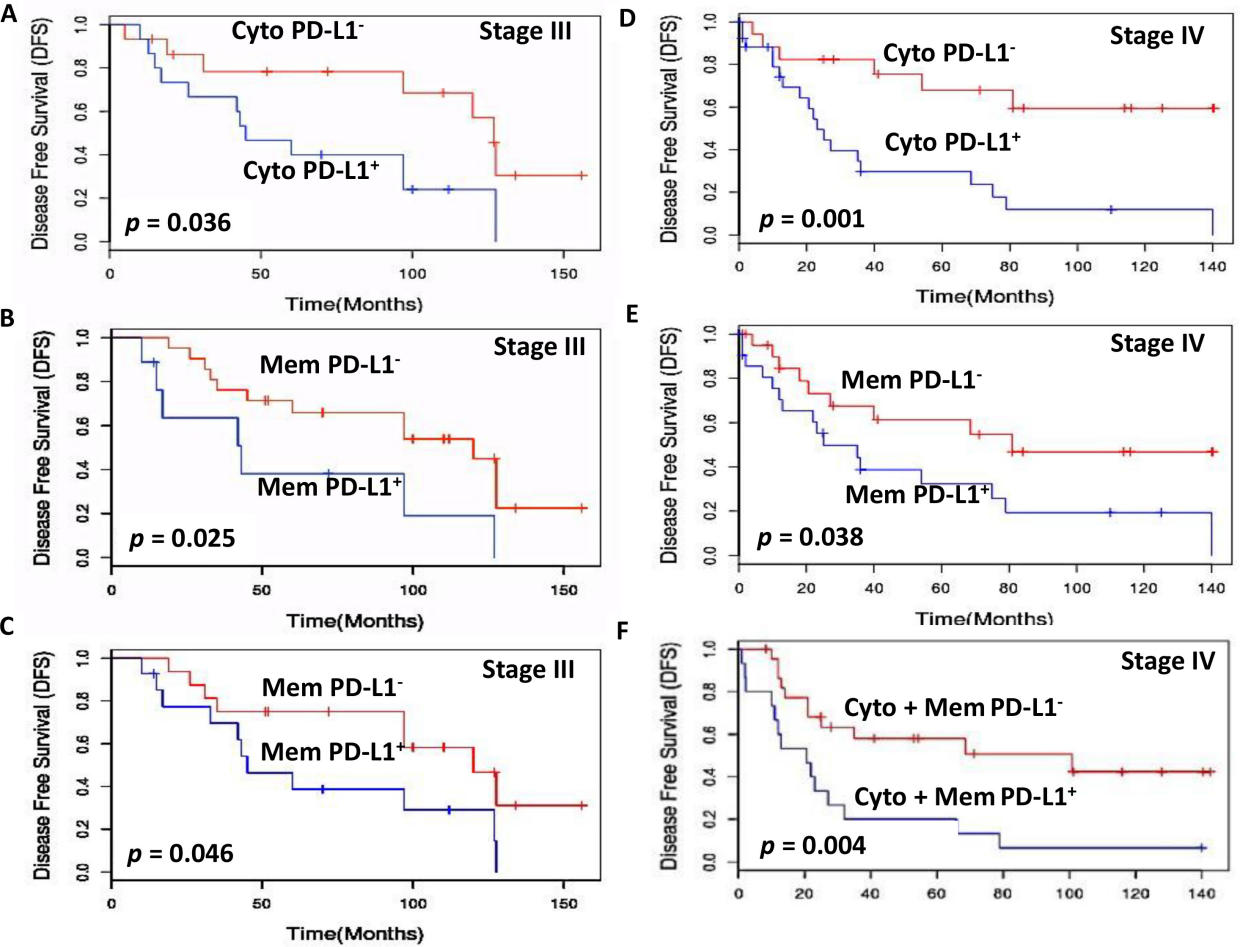
Kaplan-Meier estimation of disease-free survival in stage III and IV PTC patients Disease free survival curves showing PD-L1 expression in (**A**) Stage III cytoplasm [PD-L1 positive median disease free survival (DFS) = 53 months (blue lines) and PD-L1 negative DFS = 128 months (red lines); *p* = 0.036]. (**B**) Stage III membrane [PD-L1 positive median disease free survival (DFS) = 43 months (blue lines) and PD-L1 negative DFS = 120 months (red lines); *p* = 0.025]. (**C**) Stage III combined cytoplasm and membrane [PD-L1 positive median disease free survival (DFS) = 45 months (blue lines) and PD-L1 negative DFS = 120 months (red lines); *p* = 0.046]. (**D**) Stage IV cytoplasm [PD-L1 positive median disease free survival (DFS) = 23 months (blue lines) and PD-L1 negative DFS = 140 months (red lines); *p* = 0.001]. (**E**) Stage IV membrane [PD-L1 positive median disease free survival (DFS) = 25 months (blue lines) and PD-L1 negative DFS = 81 months (red lines); *p* = 0.038]. (**F**) Disease free survival curves showing PD-L1 expression in Stage IV combined cytoplasm and membrane [PD-L1 positive median disease free survival (DFS) = 21 months (blue lines) and PD-L1 negative DFS = 140 months (red lines); *p* = 0.004].

Further, to assess the prognostic potential of PD-L1 we compared PD-L1^+^ and PD-L1^−^ patients with matched staging and treatment history. Importantly, Stage I patients with PD-L1 cytoplasmic positive tumors had significantly shorter median DFS = 166 months as compared to those with PD-L1 negative tumors (DFS = 185 months; *p* = 0.031; Figure 3A). Stage II patients with PD-L1 cytoplasm positive tumor cells also showed significantly shorter median DFS = 62 months as compared to those with PD-L1 negative tumors (DFS = 132 months; *p* = 0.027) (Figure 3D). Comparison of the DFS in stage III PTC patients with PD-L1 cytoplasmic positive and negative tumor cells also showed significant difference (53 months vs 128 months, *p* = 0.036) (Figure 4A). Stage III PTC patients with PD-L1 membrane positive and negative tumors showed significant differences in DFS (months) 43 vs 120 (*p* = 0.025; Figure 4B). The combined PD-L1 cytoplasm and membrane positive tumor cells in stage III showed marginal significance with shorter median DFS = 45 months as compared to those with PD-L1 negative tumors (DFS = 120 months; *p* = 0.046; Figure 4C). Stage IV patients with PD-L1 cytoplasmic positive tumors had significantly shorter median DFS = 23 months as compared to those with PD-L1 negative tumor cells (DFS = 140 months; *p* = 0.001; Figure 4D). Stage IV PTC patients with PD-L1 membrane positive and negative tumors showed significant differences in DFS (months) 25 vs 81 (*p* = 0.038; Figure 4E). The combined PD-L1 cytoplasm and membrane positive tumor cells in stage IV showed significantly shorter median DFS = 21 months as compared to those with PD-L1 negative tumors (DFS = 140 months; *p* = 0.004; Figure 4F). No significant DFS difference was observed in stage I, II PD-L1 membrane alone and/or combined cytoplasmic and membrane tumor positive and negative patients.

## DISCUSSION

Impaired immune surveillance has been shown to be an important factor in enhanced tumor cell aggressiveness that results in metastasis. The aim of our study was to investigate the subcellular expression of PD-L1 in different stages of PTC and to correlate its protein expression with disease aggressiveness and outcome. Our results showed that PD-L1 expression in tumor cells correlated with aggressive PTC and was associated with poor prognosis.

Several other cancers have been reported to have increased PD-L1 expression in the plasma membrane alone or in both plasma membrane and cytoplasm [22–25]. The PD-L1 antibody used in this study is a well-established one and had been used for immunohistochemical analysis in different types of cancers by several other groups [26–31]. However in our study we observed positive PD-L1 cytoplasmic staining in all stages (I, II, III, IV) of PTC, which correlated significantly with DFS suggesting that overexpression of cytoplasmic PD-L1 can identify a subset of patients with a poor prognosis. Hence cytoplasmic PD-L1 could serve as a prognostic marker even in patients with early stage disease (Stage I and II) to stratify patients with a higher risk of residual or recurrent disease. Notably, in comparison with other cancers we observed low membrane localization of PD-L1 in stage I PTCs which gradually increased in higher stages (II and III), but was not significantly correlated with a reduced DFS. However, only in patients with stage IV disease, a significant increase in membrane PD-L1 positivity was correlated with high risk of aggressive disease, distant metastasis or death. The combined cytoplasmic and membrane PD-L1 positivity was also associated with poor prognosis. These findings suggest increased PD-L1 membrane positivity was associated with high risk of aggressive disease, distant metastasis or death. The membrane PD-L1 localization in patients with stage IV tumors also suggests these patients might respond to monoclonal anti-PD-1 immunotherapy, but further studies will be required to determine whether such patients might respond to anti-PD-L1 immunotherapy. By comparison, no such significant findings were observed in benign thyroid nodules.

In accord with previous reports [32–34], we also observed an increased expression of PD-L1 expression in chronic lymphocytic thyroiditis and Hashimoto's thyroiditis, suggesting that the chronic inflammation might provide a microenvironment enriched with different cytokines such as IFNγ, IL-1, IL-10, IL-6 that could trigger upregulation of PD-L1 expression. Hence the PD-L1 immunohistochemical staining in benign thyroid nodules with concurrent lymphocytic infiltration needs to be interpreted with caution.

In the current report a strong correlation between PD-L1 expression on tumor cells and long term prognosis has been documented in our cohort of PTCs (all stages combined). PD-L1 status has been proposed to be critical to promote tumor growth and metastasis [35, 36]. An earlier study by Cunha and his group [22] suggested PD-L1 (B7H1) upregulation in PTC may lead to blockade of the immune system based on B7H1 protein expression by immunohistochemical analysis and gene expression by real time PCR. In this study aggressive PTC patients showed higher levels of mRNA and protein than lower risk PTC but the protein expression was decreased in lymph node metastases. However, the relationship between PD-L1 (B7H1) and disease outcome by AJCC staging and subcellular distribution of PD-L1 expression was not determined in this study. In comparison, our study unequivocally demonstrated association of PD-L1 overexpression with aggressiveness and poorer prognosis based on long term follow up of these patients and Kaplan Meier and Cox regression analyses. Another recent study reported PD-1^+^ Tim-3^+^ CD8^+^ T lymphocytes showed varied degrees of functional exhaustion in patients with regionally metastatic PTC [37].

In summary, our data demonstrate that immunohistochemical analysis - based subcellular cytoplasm and membrane localization of PD-L1 expression and its association with reduced disease free survival in PTC patients, suggests its utility as a potential prognostic biomarker. This could assist in determining which PTC patients and its variants have a greater risk for a more aggressive clinical outcome in PTC management.

## MATERIALS AND METHODS

### Patients

The Research Ethics Board (REB) of Mount Sinai Hospital (MSH), Toronto, Canada, approved this study. Informed consent for scientific use of anonymized patients' data and tumor tissues was obtained from all patients (REB guideline #07-0212-E). All data were analyzed anonymously. Archived formalin-fixed paraffin-embedded (FFPE) tissue blocks from the MSH Tumor Bank were retrieved and reviewed by the pathologist. Clinical-pathological parameters were obtained from histopathological analyses and the clinical database and are summarized in Table 1. Two hundred and fifty one patients, 66 with benign thyroid nodules and 185 PTCs (median age: 45 years; range: 18–85 years) and undergoing curative surgery during the period 1992–2013 were analyzed for PD-L1 protein expression. The PTCs were staged following the guidelines of the American Joint Committee on Cancer (AJCC) stage classification system [38].

FFPE tissue sections (5 μm thickness) from these patients were deparaffinized, hydrated with series of graded alcohol and antigen retrieval for PD-L1 was performed in Tris-EDTA buffer, pH 9.0 as described by us previously [39]. After blocking the non-specific binding with background punisher (Biocare Medical, LLC, Concord, CA), the sections were incubated overnight with the monoclonal rabbit anti-PD-L1 antibody at 1:200 dilution (E1L3N, Cell Signaling Technology, Inc. (CST), Danvers, MA) and detected using The VECTASTAIN rapid protocol (Vector Labs, Burlington, ON, Canada) for immunostaining using diaminobenzidine (DAB) as the chromogen and counterstained with hematoxylin [39]. FFPE sections of Oral squamous cell carcinoma tissue were used as a positive control and thyroid cancer tissue incubated with an isotype specific IgG was used as a negative control for PD-L1 staining in each batch of immunostaining.

### Evaluation of immunohistochemistry

Immunostaining scores were based on the percentage positivity and staining intensity as described previously [39]. Sections were scored as positive if epithelial cells showed immunoreactivity in the cytoplasm and/or membrane. Percentage positive scores were assigned according to the following scale: 0 (< 10%); 1 (10–30%); 2 (31–50%); 3 (51–70%); and 4 (> 70%). Staining intensity was scored semi-quantitatively as follows: 0 (none); 1 (mild); 2 (moderate) and 3 (intense) [40]. A total score for each cytoplasmic and membrane staining was then obtained (ranging from 0 to 7) by adding the percentage positivity scores and intensity scores for each section. The IHC scoring was blinded from the histopathology report and was performed by two evaluators independently and used for subsequent analyses. The inter-observer variation between two evaluators was determined. Subsequently, ROC curves were computed to define optimal cut offs for cytoplasmic, membrane and combined cytoplasmic and membrane immunopositivity.

### Statistical analyses

The patient distribution and clinical features between PD-L1-positive and PD-L1-negative tumors were compared by chi-square test, or two-sample *t*-test as appropriate. The endpoint for this analysis was disease-free survival (DFS), which is defined as the length of time from the date of surgery on primary tumor to local or regional recurrence, distant metastasis or death. Survival curves based on PD-L1 expression were estimated using the Kaplan–Meier product-limit method with the log-rank test. Univariate Cox proportional hazards models were fit to identify factors significantly related to DFS. To assess whether the PD-L1 expression in tumor cells was an independent predictor of DFS, a multivariate Cox model was constructed to adjust for other patient/clinical characteristics that were significant in the univariate analyses. Two-way interaction terms between PD-L1 expression and other factors in the multivariate Cox model were also assessed. All analyses were two-sided, and significance was set at *p* value ≤ 0.05. Statistical analyses were performed using Sigma Plot 11 (version 11.2.0, Systat Software, Inc.), SPSS statistics software version 23 (SPSS, Chicago, IL, http://www.ibm.com/analytics/us/en/technology/spss/) and R software 3.2.2.
